# Success of a South-South collaboration on Human Resources Information Systems (HRIS) in health: a case of Kenya and Zambia HRIS collaboration

**DOI:** 10.1186/s12960-019-0342-z

**Published:** 2019-01-15

**Authors:** Victor Were, Elizabeth Jere, Kevin Lanyo, George Mburu, Rose Kiriinya, Agnes Waudo, Bwalya Chiteba, Keith Waters, Prachi Mehta, Tom Oluoch, Martha Rodgers

**Affiliations:** 1 Emory University Kenya Health Workforce Project, Nairobi, Kenya; 2 US Centers for Disease Control and Prevention (CDC), Lusaka, Zambia; 3 Division of Global HIV & TB (DGHT), US Centers for Disease Control and Prevention, Nairobi, Kenya; 4 Emory University, Atlanta, USA; 5 US Centers for Disease Control and Prevention, Atlanta, USA

**Keywords:** Human resources for health, Human resource information system, South to South collaboration, South-South collaboration, South-to-South collaboration, HRIS collaboration

## Abstract

**Background:**

Shortage of health workforce in most African countries is a major impediment to achieving health and development goals. Countries are encouraged to develop evidence-based strategies to scale up their health workforce in order to bridge the gap. South-South collaborations have gained popularity due to similarities in the challenges faced in the region. This strategy has been used in trade, education, and health sector among others. This paper is a road map of using a South-South collaboration to develop a Human Resources Information System (HRIS) to inform scale-up of the health workforce.

**Case presentation:**

In the last decade, Kenya implemented one of the most comprehensive HRIS in Africa. The HRIS was funded by the US President’s Emergency Plan for AIDS Relief (PEPFAR) through the Centers for Disease Control and Prevention (CDC) and implemented by Emory University. The Kenyan team collaborated with the Zambian team to establish a similar HRIS in Zambia. This case study describes the collaboration activities between Zambia and Kenya which included needs assessment, establishment of project office, stakeholders’ sensitization, technical assistance and knowledge transfer, software reuse, documents and guidelines reuse, project structure and management, and project formative evaluation.

Furthermore, it highlights the need for adopting effective communication strategies, collaborative planning, teamwork, willingness to learn, and having minimum technical skills from the recipient country as lessons learned from the collaboration. As a result of the collaboration, while Kenya took 5 years, Zambia was able to implement the project within 2 years which is less than half the time it took Kenya.

**Conclusions:**

This case presents a unique experience in the use of South-South collaboration in establishing a HRIS. It illustrates the steps and resources needed while identifying the successes and challenges in undertaking such collaboration.

## Background

In recent years, as several low-income countries transitioned into middle-income status, there has been a significant growth in the region. Decolonization, rising demand for equality in world affairs by developing countries, increasing political will and determination, and more equitable distribution of technical advances and resources have led to a call for more self-reliance and less reliance on high-income countries [9]. However, poor prioritization of projects, lack of accountability by donors, and limited engagement of low-income countries’ governments on development discussions are some of the challenges that need to be addressed [15]. Countries in the south are engaging in collaborative models to share innovative, adaptable, and cost-effective solutions to address development challenges [5]. This approach, known as South-South collaboration, has been recognized as a promising strategy for development since the Buenos Aires Plan of Action in 1978 [1]. The United Nations (UN) Office for South-South Cooperation describes the South-South collaboration as a “broad framework for collaboration among countries of the South in the political, economic, social, cultural, environmental, and technical domains. Developing countries share knowledge, skills, expertise, and resources to meet their development goals through concerted efforts” [18]. The high-level UN conference on South-South collaboration in 2009 provided a more comprehensive operational definition as the process whereby two or more developing countries pursue their individual and/or shared national capacity development objectives through exchanges of knowledge, skills, resources, and technical know-how and through regional and interregional collective actions, including partnerships involving governments, regional organizations, civil society, academia, and the private sector, for their individual and/or mutual benefit within and across regions [16].

Health and education are the main drivers of South-South collaboration [2]. Despite the increased use of South-South collaborations in the health domain, the extent to which the collaborations occur in service delivery, particularly outside the realm of family planning is relatively low [20]. Poor countries are often too busy dealing with their own overwhelming health problems to be able to spare staff to assist other countries [3]. Effectiveness of South-South collaborations has also been doubted due to the complexities regarding the ownership or management of the collaborations, lack of well-defined national policies, uneven shared benefits among developing countries, limited documented information on south-south success stories, resource scarcity, trade barriers, and political problems. Despite the skepticism, it is anticipated that South-South collaborations make the partner countries learn winning strategies and set their economies on the path of development [6].

Kenya and Zambia are low-income countries located in sub-Saharan Africa with an estimated population of 46 and 13 million respectively [4, 24]. The Global Health Observatory of the World Health Organization of 2012 reported that the leading cause of mortality in Kenya was human immunodeficiency virus and acquired immune deficiency syndrome (HIV/AIDS) which accounted for 14.8% of the mortalities with lower respiratory infections and diarrheal diseases at 12.3% and 6.3% respectively. HIV/AIDS was reported to account for 24.5% of the mortalities in Zambia with lower respiratory infections and malaria at 7.5% and 7.3% respectively [22, 23].

One of the greatest threats to universal provision of healthcare and attainment of health-related Sustainable Development Goals (SDG) in sub-Saharan Africa is the status of the health workforce. The WHO stated that in 2013 there was a global shortage of almost 7.2 million health care workers, with Africa accounting for 25% deficit [21]. Zambia reported having 93 health care workers (HCWs) per 100 000 population ratio while Kenya had 150 HCWs per 100 000 population ratio in the 2009 [11, 25]. Both countries’ HCWs per population ratio fall far below the WHO-recommended HCWs ratio of 230 HCWs per 100 000 population ratio required to achieve minimum service delivery coverage [12]. WHO recommended collection of reliable data and strengthening human resources for Health Information Systems (HRIS) to inform production, regulation, and deployment of health workers as a means to address the critical health workforce shortage [21]. HRIS collects and manages routine, national level, multi-cadre data on the health workforce including supply (i.e., training, exam, registration, licensure, intent to out-migrate, and continuing professional development) and deployment (i.e., health facility of deployment, date of appointment, workstation in the facility, date of promotion, disciplinary actions, date of exit, and transfers) [7]. The collected data are used by policy makers to make informed decisions on health workforce forecasting, deployment, and management [14].

## Case presentation

Kenya has one of the longest running and most comprehensive HRIS in sub-Saharan Africa. The system was featured as a best practice at the first Human Resources for Health Technical Consultation symposium in 2009 and was shortlisted by the WHO as a best practice of South-South collaboration [19]. The Kenya HRIS was funded by the US President’s Emergency Plan for AIDS Relief (PEPFAR) through the Centers for Disease Control and Prevention (CDC) and implemented by Emory University through the Emory Kenya Health Workforce Project. HRIS has benefited the regulatory bodies and the Ministry of Health through provision of accurate and timely data for decision-making, efficient regulatory services, and increased efficiency of operations. They have further reported increased re-licensing of various health professional cadres as a result of improved compliance. [7, 13, 19].

Having succeeded in Kenya, Emory University was invited to develop a similar project in Zambia. The team decided to implement the HRIS in Zambia using the Kenyan model through a South-South collaborative. The collaboration was done during the period of 2015 to 2017. An experienced Kenyan project team assisted the entire process of project implementation. This paper describes this approach and discusses the successes, challenges, and impact of the South-South collaboration during the collaboration period.

Several activities were conducted during the collaboration. These included the following:

Needs assessment: The Kenyan team conducted a comprehensive needs assessment of the two Zambian health professionals’ regulatory agencies—the Health Professionals Council of Zambia (HPCZ) and the General Nursing Council of Zambia (GNC). The assessment focused on identifying the existing gaps and developing strategies to bridge the gaps. The assessment was done in the following areas: administrative, information technology (IT) infrastructure, data management, software, and online service needs. It sought to find out data collected by the agencies, regulatory processes that have been implemented, available infrastructure, practitioners’ compliance rates, and the stakeholders among others. The assessment found out that the regulatory agencies experienced a relatively low compliance rate with little data available in electronic form. Despite having been mandated to regulate training, licensure, and practice of health practitioners, the regulatory agencies had little engagement with the health training institutions and practitioners who are key stakeholders in achieving their mandate. The findings of the needs assessment were used to develop the project work plan and approach.

Establishing the project office and team: Prior to the implementation of the project, there were no human resources on the ground in Zambia. In Kenya, a project director, two senior IT systems developers, the senior web developer, a project data analyst, and an IT consultant formed the Kenyan consulting team. The team constituted project management, programming, data analysis, and IT infrastructure skills. In collaboration with the project principal investigators and a local lawyer, the Kenyan team established a Zambian non-profit organization named Workforce Informatics and Techno Systems (WITS) in Lusaka. The local project director initiated a number of project start-up activities which included hiring other project staff needed for development of the HRIS and establishing an office in Zambia. The Zambia team was required to hold the required skills but at a lower level. The Zambia team that was established comprised of a project director, two IT systems developers, a web developer, and a data analyst.

Stakeholders’ sensitization: The Kenya and Zambia teams participated in several exchange trips to sensitize the stakeholders in Zambia and foster buy-in. These stakeholders included senior officers from the Ministry of Community Development Mother and Child Health (MCDMCH), Ministry of Health (MOH), Zambia’s two health professional regulatory agencies, and representatives from Zambia’s health training institutions among others. The Kenyan project team provided an overview of the project and its goals, demonstrated the Kenyan system, and discussed the achievements as well as challenges faced during development and implementation of the system. At the end of the orientation, key stakeholders were invited to Kenya for a more in-depth orientation and on-site visits to some of Kenya’s regulatory agencies to see the HRIS in use and interact with the users. During the site visit, the Kenya health regulatory agencies shared the HRIS implementation impact, challenges, and lessons learnt. The visit fostered buy-in since the Zambia stakeholders were able to hear about the success stories and impact of establishing a HRIS system from the beneficiaries in Kenya. In addition, the stakeholders also learnt about the challenges that they might face during the process of implementation which ensured that they were able to mitigate them early in the project implementation. The stakeholders used the lessons learnt from Kenya HRIS implementation as strategies for Zambia implementation in order to hasten the implementation period of the HRIS in Zambia. The work which was done in Kenya for 5 years took 2 years to complete in Zambia as a result of using the best practice and lessons learned from Kenya. Furthermore, the overall project cost was minimized by eliminating wastage caused by inadequate practices which were identified during the earlier implementation in Kenya.

Technical assistance and knowledge transfer: The Kenya project team provided the Zambia project team with one-on-one orientation and training as well as consultation. The technical team shared their skills, knowledge, and tips on the various areas of implementation through technical assistance. These were done through Internet-based calls, emails, and face-to-face consultations. Furthermore, the Kenyan team undertook evaluations of the Zambian technical team’s work products and outputs and provided advice and constructive critiques where necessary. The Zambian team managed and implemented the project taking into consideration the differences in some of the social, technical, economic, and environmental differences between the two countries to ensure a successful implementation of the project.

Software reuse: Software development of any information system begins with an assessment and analysis of the business functions of the agencies which outline the information needs and software requirements. The Kenya team assisted the Zambian team in conducting a business process analysis of the two Zambian councils. The functions in the system were based on these business processes as well as the standard operating procedures to carry out daily tasks. Due to the similarities of the functions of the councils in Kenya and Zambia, the data model and software developed for the Kenyan HRIS was easily used in Zambia with minimal customization by the Zambia project programmers. The Zambian programmers were invited for a week-long training in Nairobi on the Kenya software, and their work plans for customizing the software for Zambia were developed in collaboration with the Kenya team. The Kenya team then held regular Internet-based calls and emails with the Zambian technical team and remotely reviewed codes submitted by the Zambian programmers from time to time.

Documents and guidelines reuse: The Kenyan team shared several project documents and guidelines that were customized and adopted by the Zambia team. These documents included business process analysis guidelines, standard operating procedures templates, sustainability plans, gap analysis guide, implementation plan templates, and user manuals among others.

**Fig. 1:**
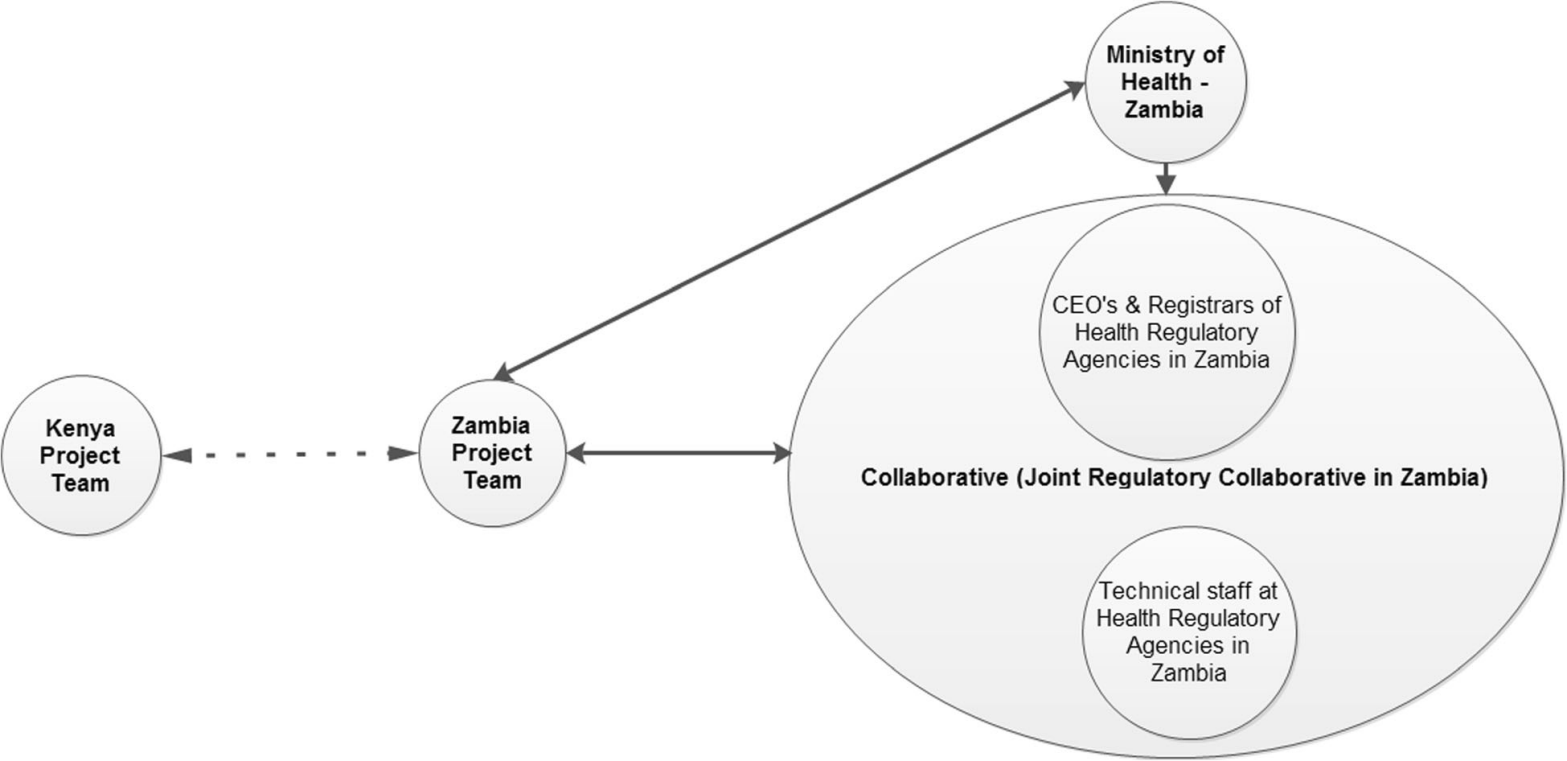
Project structure

Project structure and management: The concept of a Joint Regulatory Collaborative (JRC), as proven successful in Kenya, was adopted in Zambia (Fig. 1). This entails a consultative forum comprising registrars and chief executive officers of various regulatory agencies and the respective Ministry of Health (MOH). JRC also included a subcommittee of technical staff referred to as the Joint Technical Advisory Committee (JTAC). Generally, most regulatory agencies are fairly independent of each other despite their functional similarities, and collaboration was rare. Establishment of the JRC provided a forum for these agencies to collaborate, share ideas, and realize synergies. For example, as both countries’ regulatory agencies are eager to improve services by the development of an online portal connected to the HRIS which allows professionals to obtain service online, issues such as the need for expensive hosting of website portals were identified as a challenge and addressed through joint hosting of common services. The collaboration assisted the agencies to cost-share and innovate. On project management, the Zambian project was managed independently by internal project management, who also ensured that project work plans and budgets were developed and activities therein implemented and monitored according to the work plans. The Kenyan team only provided technical oversight. Furthermore, since most of the work of establishing a new project was carried out collaboratively between the Zambian and the Kenyan team, Emory University’s faculty time and travel were minimized with 80% of the budget going to Africa. As a result, most of the funding was used to do the actual work of the project implementation. In addition, use of the Kenyan consultants resulted in the project incurring cheaper consultancy cost compared to using expensive overseas consultants.

Project formative evaluation: To ensure successful implementation of the project in Zambia, the Kenyan team conducted a midterm implementation review of the HRIS as well as data quality review. The reports from the reviews were used to inform areas that required improvement after the initial rollout of the system. The reports were circulated to both the councils and the Zambia project team.

The HRIS system was fully implemented in the two existing health professional regulatory agencies in Zambia. Some of the functionalities that were being used include student tracking, professional registration, professional licensure, and health facilities licensure. Currently, the regulatory agencies are finalizing rollout of the regulatory online services. As a result of the successful rollout of the system, the Zambia health professional agencies are reporting several impacts: increase in health professional licensure compliance, efficiency in delivery of regulatory services with most of the service client waiting time having being reduced, and timely generation of accurate HRH reports to the MOH among others.

Despite the successful collaboration, several challenges were experienced during the initiative. Firstly, it is important to anchor any development project on existing government policy and other environmental factors. The diversity of the government policies in the two countries highlighted this issue since some of the best practices from Kenya could not be transferred directly to Zambia. For example, students in Kenya are required to submit their documents for verification to their regulatory agency after they have been admitted to a training institution. In Zambia the students are required to have their qualifications document verified by their regulatory agency before they are admitted to a training institution. The differences in the technology development between the two countries affected the adoption of some of the technologies used in Kenya, e.g., financial transactions through mobile phones which were not as mature in Zambia as opposed to having been used widely in Kenya. This posed a problem in the implementation of Kenya online services which utilized mobile phones as a means of making payment to online services. Furthermore, differences in national and public holidays also hindered some meetings and affected project timelines.

The technical expertise level difference between the two teams as a result of varying experiences was also an issue. The Kenya team had more experience in HRH and system development while the new Zambia team had limited knowledge on HRH and system development. These differences resulted in longer and more consultative sessions to ensure correct implementation. Lastly, the Kenya team faced time constraint challenges due to competing tasks as they were also implementing the Kenyan project. This required the Kenyan team to create more time from their busy schedules to support the Zambia project.

Several lessons were learned during the collaboration by both teams:

1. Communication—People from different countries and/or cultures have different communication styles, varying customs and expectations, and differing levels of communication infrastructure. To minimize communication barriers, partners should be willing to use any and all forms of communication, including phone and e-mail. In our experience, Internet-based calls proved most useful for prompt exchange of information and was sometimes more reliable than e-mail. The teams scheduled a weekly conference call which acted as consultative sessions. During the communication, Zambia and Kenya teams designated one person who was the main point of contact between the teams.
2. Planning—Advance planning was key to smooth running of the project. To assure appropriate collaboration development of a joint work plan where both teams factored the time needed for collaboration activities (e.g., meetings, travel) was critical. The joint work plan ensured that no project implementation activity lagged behind due to unavailability of either team.
3. Promotion of team cohesion among the collaborating organizations by building informal time into exercises or projects and organizing regular meetings was important in building trust and identifying each other’s strengths and weaknesses. The teams were also open-minded to better accommodate the other team’s views for the benefit of successful project implementation.
4. Development of a calendar displays each partner organization’s national and religious holidays, team members’ vacations, and other absences. This helped in scheduling travel and establishing project timelines.
5. Willingness to learn and commitment ensured the knowledge transferred is mastered and appropriately applied to the current projects as well as used to innovate for the future.
6. The recipient country’s project team should possess the minimum experience, skills, and knowledge (both technical and administrative) required for successful project implementation, taking into consideration the different country’s environmental factors.

## Discussion

**Fig. 2:**
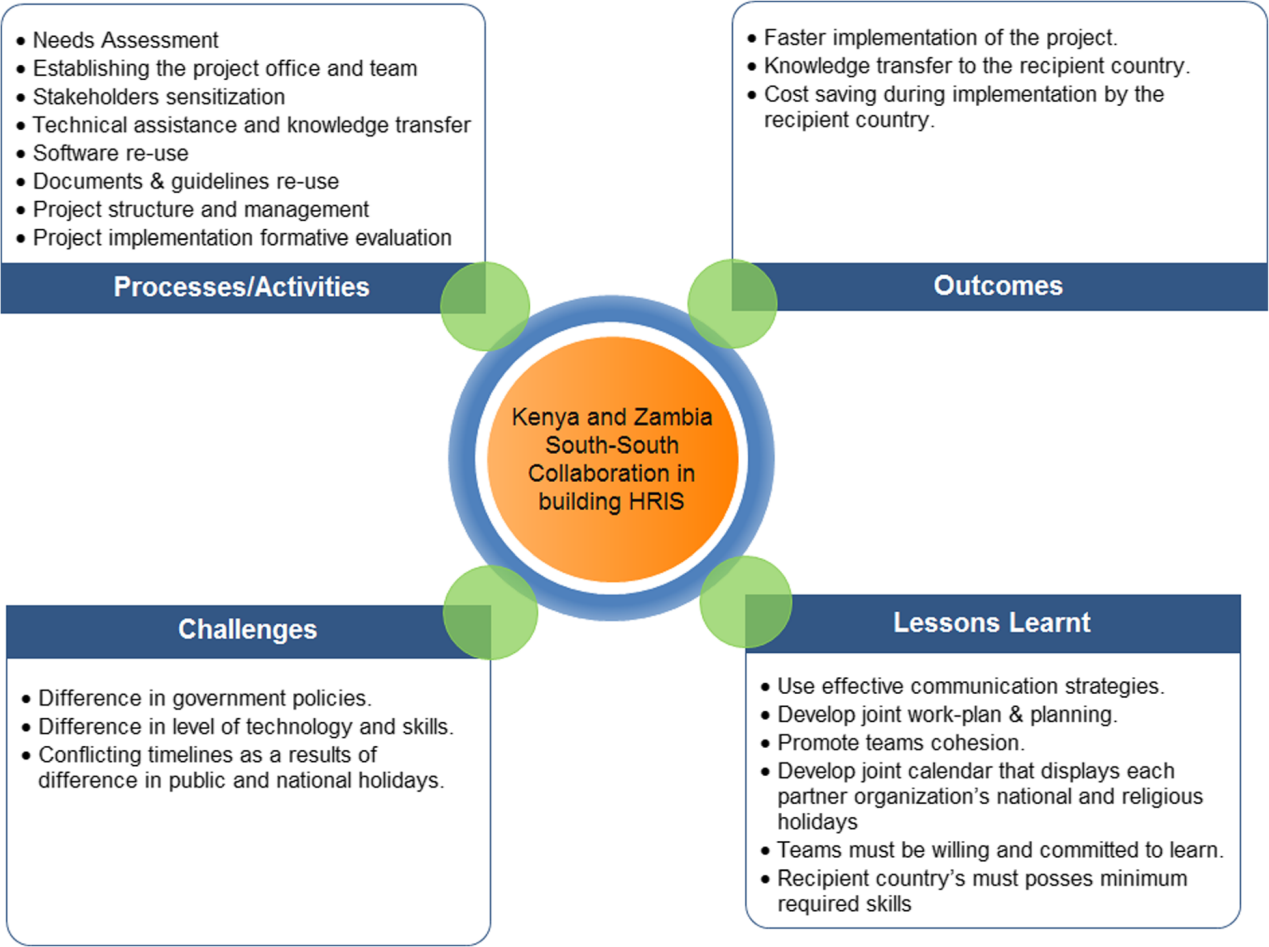
Collaborative model

The South-South collaboration described resulted in a cost-effective and efficient way for establishment of the HRIS system in Zambia (Fig. 2). The approach used in this project may be technically considered a triangular cooperation, meaning that a traditional donor country (in this case, the United States of America) facilitates South-South collaboration through provision of funding, training, and management systems. However, in the HRIS case, funding was provided by CDC and only minimal administrative, technical, and scientific assistance was provided by Emory University.

US Government’s global health programs, such as PEPFAR, generally hire a combination of American Universities and profit/non-profit US organizations that provide international development primarily through United States Agency for International Development (USAID), CDC, and other federal US agencies. Many of these organizations establish in-country offices and infrastructure to manage funded projects. The in-country offices are largely staffed by local residents and supported by sponsoring agencies staff members that are either assigned to work in these countries or travel from overseas several times a year. Although this model works well, its drawbacks are that a significant proportion of the funding stays with the sponsoring agencies and their staff, and might create the perception that only overseas personnel have the knowledge to run development projects. South-South collaboration fosters self-reliance by giving local experts a chance to take on a higher level of responsibility compared with North-South collaborative approaches. Using a South-South approach which supports collaboration and knowledge transfer from one local project team to another gives local teams the experience, responsibility, and ownership that is necessary to sustain development in their countries. This model enables the local teams to go beyond their traditional role as recipients of expertise and knowledge transfer [8, 10].

South-South collaboration has the potential to reduce costs. Indeed, the United Nations Environment Programme (UNEP) reports that “South-South cooperation can increase the flow of information, resources, expertise and knowledge among low-income countries at reduced costs” [17]. Significant cost reductions were realized by using local regional consultants rather than importing consultants from the donor country resources at higher cost.

Modern and inexpensive communication methods such as Internet-based calls and email have allowed for distance communication and exchange of materials, making this type of collaboration possible and essential. The UN Office for South-South Collaboration emphasizes that this type of collaboration increases and improves communications among low-income countries, leading to a greater awareness of common problems and wider access to available knowledge and experience as well as the creation of new knowledge in tackling development problems [18]. Indeed, the mature Kenya project has hosted many delegations from other African countries who are interested in developing regulatory HRIS. In the spirit of triangular cooperation, CDC has facilitated such exchanges between the Kenya project and other African countries which include Tanzania, Sierra Leone, Uganda, and Nigeria.

The similarities and differences between the health professional regulatory systems in both Kenya and Zambia, as well as the limited IT infrastructure of regulatory agencies in both countries, were well understood by both teams, allowing for an exchange of lessons learned and problem-solving that were transferable between two developing countries. A report by Task Team on South-South Cooperation pointed out that due to similar development levels and experiences, low-income countries can share good practices and develop solutions that are highly adaptable to local economic and social conditions [8].

Using the South-South approach allowed Zambia to develop and implement HRIS at a much faster pace by customizing already developed software and materials, lessons learned, and expertise. Since the software was developed using US federal government funds, it could be easily shared between countries as it becomes public domain software. Similarly, the Kenyan team also sharpened their project management skills through the collaboration. In addition, the Kenya team is in the process of producing documents and other materials that can be shared for use by other countries. They are also open to providing a similar consultancy to other countries through expansion of their role in this type of collaboration.

## Conclusion

We describe a successful South-South collaboration between two project teams, from Kenya and Zambia, triangulating with Emory University and CDC, to develop and implement a HRIS. Many other African countries have similar needs for HRIS that can provide data on the health professional workforce to address the many problems and issues associated with scale-up and strengthening of the health workforce in sub-Saharan Africa. This South-South approach could be utilized effectively by other countries to more quickly develop and implement their own HRIS. The collaboration further shows evidence-based best practice of South-South collaboration to inform and influence development cooperation at the global level.

## Abbreviations

AIDS: Acquired immunodeficiency syndrome
CDC: Centers for Disease Control and Prevention
DGHT: Division of Global HIV & TB
GNC: General Nursing Council of Zambia
HCWs: Health care workers
HIV: Human immunodeficiency virus
HPCZ: Health Professions Council of Zambia
HRH: Human Resources of Health
HRIS: Human Resource Information System
IT: Information technology
JRC: Joint Regulatory Collaborative
JTAC: Joint Technical Advisory Committee
MCDMCH: Ministry of Community Development Mother and Child Health
MOH: Ministry of Health
PEPFAR: President’s Emergency Plan for AIDS Relief
PPD: Partners in Population and Development
SDG: Sustainable Development Goals
TB: Tuberculosis
US: United States
UN: United Nations
UNEP: United Nations Environment Programme
USAID: United States Agency for International Development
WHO: World Health Organization
WITS: Workforce Informatics and Techno Systems
